# Recognized and unrecognized dural punctures in 12,981 labor epidurals: an audit of management

**DOI:** 10.1007/s00540-022-03062-7

**Published:** 2022-04-27

**Authors:** Victoria A. Eley, Wally Abeypala, Andrea Kelley, Nihal Kumta, Adrian Chin

**Affiliations:** 1 Department of Anaesthesia and Perioperative Medicine, The Royal Brisbane and Women’s Hospital, Butterfield St, Herston, Brisbane 4006 Australia; 2 Faculty of Medicine, The University of Queensland, St Lucia, Brisbane 4067 Australia

**Keywords:** Dural puncture, Epidural blood patch, Post-dural puncture headache, Labor analgesia, Obstetric

## Abstract

**Purpose:**

Unintentional dural puncture (DP) and post-dural puncture headache (PDPH) continue to cause discomfort and disability in a small proportion of post-partum women. We report an audit of the management of recognized and unrecognized DP over 10 years.

**Methods:**

Clinical data were prospectively collected for women who experienced a recognized DP or developed symptoms following a neuraxial procedure. Details were documented regarding patient characteristics, the neuraxial procedure, symptoms reported, and epidural blood patches. We reported rates of recognized DP, unrecognized DP, PDPH, and blood patches performed. Data were presented as number (percent) and proportions of interest compared using Chi square analysis.

**Results:**

Between January 2009 and December 2018, 12,981 women utilized labor epidural analgesia. A recognized DP occurred in 131 (1.0%) and an unrecognized DP in 60 (0.5%), with unrecognized DPs comprising 31% of the total. Of 131 recognized punctures, 86 (66%) developed a PDPH. A total of 146 (1.1%) women experienced a PDPH. Of those, a blood patch was performed in 93 (64%). Intrathecal catheters were inserted for > 24 h in 43 (33%) women with a recognized DP. Of those, 33 (77%) developed a PDPH, compared to 53 (60%) of those without an intrathecal catheter in situ for > 24 h (*P* = 0.06).

**Conclusions:**

Rates of DP were consistent with those reported by others. Unrecognized DP comprised a third of all DP, and systematic post-neuraxial follow-up is essential to identify these women. Epidural blood patch was performed in most women experiencing symptoms of PDPH.

## Introduction

Unintentional dural puncture (DP) remains a common and costly complication of obstetric anesthesia, affecting between 0.15 and 3.5% of obstetric neuraxial procedures [1–6] Following DP with an 18- or 16- gauge Tuohy needle, a post-dural puncture headache (PDPH) follows in approximately 50% of women. This headache is commonly severe [4], and although self-limiting, [7] causes significant distress and disability in post-partum women. PDPH has also been associated with serious complications such as cerebral sinus thrombosis and subdural hematoma [8].

In the previous 20 years, there have been significant practice changes in the procedure of epidural insertion and techniques to prevent and successfully manage PDPH. Identification of the epidural space by loss of resistance to saline is now more common than loss of resistance to air [4, 9]. The combined spinal-epidural (CSE) [10] technique is increasingly used in the labor ward setting, as is pre-procedural ultrasound [11]. Infusion of normal saline into the epidural space and prophylactic blood patching to prevent the development of PDPH have not been supported by high-level evidence [12, 13]. Insertion of an intrathecal catheter to provide ongoing analgesia has practical benefits; however, the effect on PDPH prevention is less clear [9, 12, 14]. Sphenopalatine ganglion block for treatment of PDPH is an attractive, non-invasive option compared with the gold standard epidural blood patch, however evidence of efficacy is lacking at the current point in time [12].

Assessing interventions to prevent or treat PDPH will remain challenging due to the small proportion of women affected and the natural history of resolution of the condition. Therefore, reporting the rates, management, and outcomes of dural puncture following neuraxial analgesia with a Tuohy needle is important for benchmarking and comparison of techniques and outcomes. In this audit, we report the management and outcomes of dural puncture from Tuohy needle insertion for labor analgesia at a single tertiary Australian institution.

## Methods

We obtained exemption from full ethical review (LNR/2021/QRBW/73681) to undertake a retrospective analysis of prospectively collected data. The Royal Brisbane and Women’s Hospital (RBWH) is a tertiary referral center providing care for 4000–5000 annual deliveries. As a referral center with a neonatal intensive care unit, the hospital cares for mothers with complex co-morbidities, complications of pregnancy, and pre-term neonates. The RBWH is a major teaching hospital and provides obstetric anesthesia training for trainees in years one to five of a five-year training program. The labor epidural rate ranges from 30 to 34%. We have previously reported that trainees perform approximately 85% of labor epidurals, with specialist staff performing epidurals in more complex patients [15].

The audit period extended from January 2009 to December 2018 and documented the clinical care of women with a recognized DP and of those with post-epidural headaches and symptoms diagnosed as a PDPH (a suspected unrecognized DP). Whenever there was a recognized DP, the treating physician documented the details of anesthesia care on a hard-copy datasheet and arranged clinical follow-up. These data sheets were also completed for women with a suspected unrecognized DP. Lead clinicians (WA and AK) were responsible for the manual input and storage of this information electronically via a Microsoft Excel spreadsheet. If the information on the data sheet was insufficient, additional information or context was obtained from the clinical record. The audit data reported was restricted to neuraxial techniques performed at our institution for the purposes of labor analgesia. Patients who had their neuraxial technique performed at a different institution but had their headaches managed at our institution were not included. Headaches associated with neuraxial techniques for procedures other than labor analgesia were not included.

The recorded information included the patient’s age, parity, pre-pregnancy body mass index (BMI), the gauge of the Tuohy needle used for insertion, and whether an epidural or CSE technique was used. If an intrathecal catheter was left in situ, the period of time was documented; either less than or greater than 24 h. Clinical details regarding follow-up included the presence of headache and the nature of accompanying symptoms (tinnitus, photophobia, neck pain, or stiffness). The diagnosis of a PDPH was a clinical diagnosis and the lead clinicians referred to the International Classification of Headache Disorders (ICHD), 3rd edition, to help resolve uncertainties [7]. A recognized DP was considered to have occurred when cerebrospinal fluid flowed from the Tuohy needle or was freely aspirated from the epidural catheter. A recognized DP was also considered to have occurred based on clinical assessment following the administration of drugs to the catheter, e.g., hypotension or level of block out of context with the dose and volume of the administered drug. If an intrathecal catheter was documented as having been inserted, this was considered to be a recognized DP. An unrecognized DP was considered to have occurred when there were none of the above criteria; headache symptoms were consistent with a PDPH and, in accordance with the ICHD, were “not better accounted for by another ICHD-3 diagnosis” [7]. Details of PDPH management included whether an epidural blood patch was performed, the number of blood patches performed for each patient and the volume of blood utilized in each case.

During the audit period, the usual, but not mandatory, neuraxial technique performed on the birth suite was epidural analgesia. The equipment used was the Portex™ Tuohy needle (Smiths Medical Australasia, Macquarie Park, NSW, Australia), in the 16 or 18-gauge size. Previous data have demonstrated that neuraxial analgesia in our institution is performed predominantly with the patient in the sitting position [15] and using the loss-of-resistance technique to saline rather than air. Low concentration anesthetic solutions were utilized, with the institutional pre-mix consisting of 0.1% bupivacaine with fentanyl 2mcg/mL. Following a recognized DP, it was not departmental practice to infuse normal saline into the epidural space, nor to perform a prophylactic epidural blood patch.

When reviewing a woman with a presumed diagnosis of PDPH, the usual approach of the specialist anesthetist was to offer hydration, oral analgesia, and if this conservative management was unsuccessful, an epidural blood patch (EBP) was offered from any time > 48 h following the presumed or documented dural puncture. Consistent with peer-reviewed recommendations [13], an EBP was offered based on the intensity of maternal symptoms and the impact on their daily activities. EBP was only undertaken after a full discussion of the risks, benefits, and alternatives and with the patient’s consent.

Some practice changes occurred during the audit period. From 2009, when a recognized unintentional DP occurred, some clinicians left the epidural catheter in the intrathecal space, to provide analgesia and with the secondary aim of preventing the development of a PDPH. This was not a documented department protocol. The alternative action was to remove the intrathecal catheter and re-site an epidural at a different lumbar interspace. From 2016, sphenopalatine ganglion blocks were offered and performed by some clinicians, but this was not considered standard practice.

Our acute pain service ensures the follow-up of all women following neuraxial procedures for obstetric care. Staff from nursing, midwifery, and medical specialties are encouraged to refer post-partum women with symptoms suggestive of neuraxial complications for anesthesia review. On discharge, a direct phone number is provided for women to seek advice or assessment regarding post-neuraxial symptoms of concern, including headache.

We aimed to provide answers to the following questions relating to patients cared for during the audit period:

1. What proportion of women having neuraxial analgesia with a Tuohy needle had the procedure complicated by a recognized unintentional dural puncture?
2. What proportion of women with a recognized DP subsequently developed a PDPH?
3. What proportion of women having neuraxial analgesia using a Tuohy needle were diagnosed with PDPH?
4. What proportion of women experiencing a PDPH were administered a blood patch?
5. What was the frequency with which the different symptoms of PDPH were reported?

Categorical data are presented using numbers and percentages, and continuous data by means and standard deviations or median and interquartile ranges where appropriate. Proportions of interest were compared using the Chi square test or Fishers Exact Test where appropriate. *P* less than 0.05 was considered significant. Analysis was undertaken using Microsoft® Excel® for Microsoft 365 MSO (Version 2111).

## Results

**Fig. 1:**
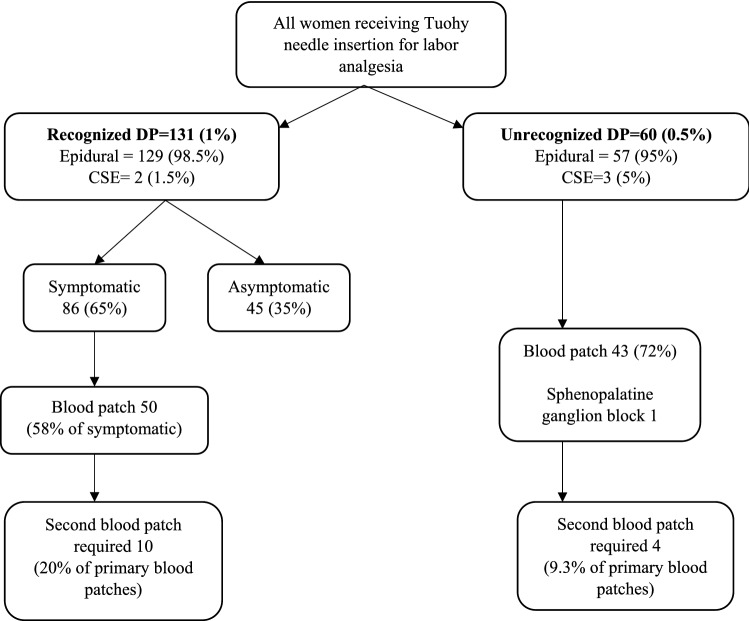
Management of women experiencing a recognized or unrecognized unintentional dural puncture (DP) following Tuohy needle insertion, Royal Brisbane and Women’s Hospital, January 2009- December 2018. *CSE* combined spinal-epidural

During the audit period, 12,981 women received neuraxial labor analgesia on the birth suite. In 131 (1.0%, [95%CI 0.8–1.2%]) women a recognized DP occurred and an unrecognized DP became apparent in 60 (0.5%, [95%CI 0.4–0.6%]) women, affecting 191 women in total (1.5%, [95%CI 1.3–1.7%]). Symptoms of PDPH occurred in 86 (66%, [95%CI 57–74%]) women with a recognized DP, thus 146 (1.1%, [95%CI 1.0–1.3%]) women experienced PDPH symptoms. Of the 191 DPs, 60 (31%, [95%CI 25–39%]) were unrecognized. Figure 1 shows the type of neuraxial analgesia performed and the management of all women with a recognized or unrecognized DP with a Tuohy needle. The characteristics of the 191 women with recognized and unrecognized DPs are shown in Table 1.

**Table 1:** Characteristics of 191 women experiencing recognized and unrecognized dural punctures, Royal Brisbane and Women’s Hospital 2009–2018

Characteristic	Result
Age, years mean (SD) *n* = 189	28.8 (5.2)
Gestation, weeks median (IQR) *n* = 189	39.6 (38–40)
Nulliparous n(%) *n* = 189	133 (70)
Body mass index^a^ kg/m^2^ median (IQR), *n* = 178	24.5 (21–29)
< 18.5 *n* (%)	13 (7)
18.5–23.9 *n* (%)	69 (39)
24.0–29.9 *n* (%)	54 (30)
30–39.9 *n* (%)	36 (20)
≥ 40 *n* (%)	6 (3)

^a^Pre-pregnancy body mass index

All women who were symptomatic either from a recognized or unrecognized dural puncture experienced a headache. The rates of other symptoms are presented in Table 2. Thirteen of the 146 symptomatic women (8.9%, [95%CI 4.8–15%]) underwent imaging of the brain with computed tomography or magnetic resonance imaging. The only central nervous system abnormality detected on imaging was bilateral subdural hygromas, with no associated sub-dural hematoma, in one patient. This patient had an unrecognized DP and received two epidural blood patches, utilizing 22 and 32 mL of autologous blood, respectively. The imaging was performed after the second blood patch.

**Table 2:** Symptoms of women with a recognized dural puncture compared with women with an unrecognized dural puncture. The Royal Brisbane and Women’s Hospital January 2009- December 2018. Number (percent) shown, with 95% confidence intervals

Symptom	Symptomatic women with a recognized dural puncture *n* = 86	95% Confidence interval (%)	Unrecognized dural puncture *n* = 60	95% Confidence interval (%)	*p* value
Headache	86 (100)	NA	60 (100)	NA	NA
Neck Stiffness	49 (57)	47–67	39 (65)	53–77	0.33
Photophobia	22 (26)	16–35	28 (47)	34–59	0.01
Tinnitus or blocked ears	20 (23)	14–32	14 (23)	13–34	0.64

*NA* Not applicable

Of the 191 women who experienced a recognized or unrecognized DP, in 98 (51%, [95%CI 44–59%]) the epidural was performed using an 18-gauge Tuohy needle and in 89 (47%, [95%CI 39–54.0%]) using a 16-gauge Tuohy needle (data missing in 4 patients). In the 131 recognized DP, 59 (45%, [95%CI 36–54%])) were performed using a 16-gauge Tuohy needle (3 missing). In the 60 unrecognized DP, 28 (47%, [95%CI 34–60%]) were performed using a 16-gauge Tuohy needle (1 missing).

A blood patch was performed in 50 of the 86 symptomatic women with a recognized DP (58%, [95%CI 47–69%]) and in 43 of the 60 women with an unrecognized DP (72%, [95%CI 59–83%]). Of all 146 symptomatic women, a blood patch was performed in 93 (64%, [95%CI 55–72%]). A sphenopalatine ganglion block was undertaken in one woman with an unrecognized DP, and she did not subsequently receive a blood patch.

Insertion of an intrathecal catheter was the only prophylactic measure performed in those DPs that were recognized on insertion. In those 131 women with a recognized DP, an intrathecal catheter was inserted in 70 (53%, [95%CI 44–62%]); of these, 43 remained in-situ for > 24 h and 27 remained in-situ for < 24 h. In the 43 women in whom the intrathecal catheter remained in situ for > 24 h, 18 (42%, [95%CI 27–58%]) received a blood patch. In the 88 women who did not have an intrathecal catheter inserted, or in whom it remained for < 24 h, 32 (36%, [95%CI 26–47%]) received a blood patch, and this difference was not statistically significant (*χ*^2^ = 0.37, *P* = 0.54). Thirty-three women (77%, [95%CI 61–88%]) women experienced PDPH symptoms when the catheter was left in situ for > 24 h, compared with 53 (60%, [95%CI 49–71%]) of the 88 women who did not have an intrathecal catheter inserted, or in whom it remained for < 24 h. This difference was not statistically significant (*χ*^2^ = 3.50, *P* = 0.06). The mean (SD) volume of blood injected in the epidural space was 22 mL (4.7) with a range of 10–32 mL. A repeat blood patch was performed in 14 women, comprising 15% of all women receiving an initial blood patch. No women received more than two blood patches.

## Discussion

Our results are within the range reported in the literature, with 1.5% of women who received epidural or combined spinal epidural analgesia for labor experiencing either a recognized or unrecognized DP. The proportion of women developing symptoms following a recognized dural puncture was 65% and similar to that reported by others [2]. Overall, 1.1% of patients were diagnosed with PDPH. A single epidural blood patch was administered in the majority of patients, with only 15% receiving a second patch, similar to that reported by Van de Velde et al. [2] Neck stiffness and photophobia were the most common symptoms accompanying headache. The women who experienced these complications were mainly at term and nulliparous. The majority of women were in the normal or overweight categories of BMI. There were also women at both extremes of BMI, consistent with technical challenges for anesthetists at both ends of the spectrum.

Our results highlight the importance of patient follow-up after neuraxial labor analgesia, with 31.4% of DPs remaining unrecognized until symptoms developed post-partum. Similar rates have been reported by Paech et al. [4], and Van de Velde et al. [2], however other audits have not reported unrecognized DP [5]. Unrecognized DP rates from over 43,000 women in Singapore were only 7.9% of all DPs, and this study also reported extremely low overall DP rates (0.15%) and very low rates of blood patching (9.5% of those with PDPH) [1]. Our active patient follow-up system contributed to the accurate identification of recognized and unrecognized DP and those that became symptomatic after discharge from the hospital. This system utilizes existing staff and communication systems with little additional cost, facilitating near-complete detection of post-neuraxial complications. Failure to follow women and detect symptoms of unrecognized DP may lead to an underestimation of the true rate of PDPH. This may also indicate that women could be suffering, seeking non-specialized help elsewhere, and potentially missing out on available treatment options [8, 12].

A recent retrospective analysis of over 1 million post-partum women in the United States suggested that cerebral sinus thrombosis and sub-dural hematoma may occur in up to 1:320 women experiencing a PDPH [8]. Postnatal depression, headache, and low back pain were also associated with PDPH. Brain imaging was not commonly undertaken in our institution and overall revealed only one central nervous system abnormality, subdural hygromas, which were likely to be associated with the unrecognized DP. While treatment with an epidural blood patch has not been demonstrated to reduce serious neurological complications, follow-up to permit the diagnosis of neurological sequelae following neuraxial procedures is clearly indicated [16].

When a DP is recognized during insertion for labor analgesia, the (usually urgent) requirement for analgesia remains. This may be the reason that intrathecal catheter insertion following DP is increasing in popularity. Evidence for the effectiveness of an intrathecal catheter in preventing PDPH development continues to evolve [5, 9, 14, 17, 18]. The most recent meta-analysis (including a trial sequential analysis) did not show a reduction in PDPH when intrathecal catheters were left in-situ [19]. Despite this, the intrathecal catheter does provide a route for analgesia provision without re-siting the epidural. Subsequent analgesia, or anesthesia if urgently required, can be rapidly and reliably established. The requirement for frequent clinician attendance for the duration of labor is a disadvantage of this approach. In our cohort, an intrathecal catheter was inserted in just over a half of cases where DP was recognized. This indicates that anesthetists’ opinions were evenly divided as to the best course of action. In some centers, this approach has been protocolized within the institution [2], in others the choice is left to the treating clinician [1]. The small numbers analyzed in our audit suggest no influence of intrathecal catheter placement on the incidence of PDPH or the requirement for an epidural blood patch.

There are many factors that influence variation in DP and PDPH rates. These include changing work patterns of specialist anesthetists and the allocation of more junior anesthesia trainees to our institution, which occurred halfway through this 10-year period. When reporting the clinical experience of DP, it is important to consider the relevance of the different numbers and percentages. For instance, the number and percentage of recognized and unrecognized DP could be considered a reflection of epidural insertion technique, or the institutional level of experience/supervision. Conversely, the number and percentage of PDPH are important in terms of the patient experience. A dural puncture identified on insertion is of little consequence to a post-partum woman if she remains asymptomatic.

There are limitations to our audit. Reporting the practice of a single center, our outcomes may not be generalizable to other institutions. As data were collected over a 10-year period, changes in practice over time will have influenced the findings, and the data is incomplete in parts. We have not reported the patient position during insertion, the seniority of the proceduralist, or the number of attempts during insertion. Therefore we have been unable to identify important risk factors for recognized or unrecognized DP in this cohort. While we have reported the pre-pregnancy BMI, we have not reported the BMI at delivery, which is arguably of more relevance to these data. BMI category may influence the rate of DP and high BMI may attenuate the development of PDPH [20]. We have not reported the severity or duration of headache symptoms at diagnosis or after performing an epidural blood patch. The strengths of our data include the detailed description of intrathecal catheter use, the description of symptoms reported by the women affected by recognized or unrecognized DP and the active identification of unrecognized DPs.

## Conclusions

Our audit demonstrates a fluctuating rate of recognized, unrecognized DP and PDPH over a 10- year period. Over that time, unrecognized DP remained a constant feature, comprising around a third of all DPs. Despite the consistent occurrence of PDPH over the years, imaging of the brain occurred in a small proportion of women, and only one abnormality was detected. Intrathecal catheter use was not associated with a reduction in epidural blood patch requirement but is likely to have been a convenient method of providing analgesia. We recommend active follow-up of women following epidural analgesia, to allow rapid diagnosis and provide access to epidural blood patching, which has a high success rate in treating PDPH. Evidence to support the use of less invasive treatments in the future would be welcomed.
